# Current Hospital Topics

**Published:** 1908-01-11

**Authors:** 


### HOSPITAL ADMINISTRATION.

### CONSTRUCTION AND ECONOMICS.

### CURRENT HOSPITAL TOPICS.

**Sir Malcolm Morris, K.C.V.O.**

Sir Malcolm Morris will be congratulated by his many friends upon the honour which has been conferred upon him by the King. For twenty years, as surgeon to the skin department of St. Mary's Hospital, he rendered excellent service, and during the whole of that time he interested himself in the extension and administration of the hospital. Sir Malcolm is a man of great common sense, good organising powers, and has a gift of writing, which made the revised and new series of *The Practitioner* a few years ago, one of the best written and most interesting of medical periodicals. Sir Malcolm has never really received the recognition which was due to him in connection with the enormous amount of work which he lavished upon the National Association for the Prevention of Consumption. This movement attracted Sir Malcolm, and he threw himself into the work with an energy and a zeal which gave rise to some feelings of jealousy, and so tended to withhold from him his due meed of praise for services which made the whole community his debtor. We hope that Sir Malcolm Morris will long live to enjoy his honours and to continue the useful work with which he has identified himself in many directions.

**Dr. George F. Shrady.**

It is with feelings of deep personal regret that we draw attention to the work of the late Dr. George F. Shrady, whose lamented death was briefly noticed in our issue of the 4th instant. Dr. Shrady was the founder of the *New York Medical Record*, of which he was chief editor for the long period of thirty-eight years. Dr. Shrady made the *Medical Record* of New York the foremost of American medical journals, and his genial personality, and the courage he displayed in dealing with many questions of grave importance to the public, should win for him the grateful recognition of the American people. Dr. Shrady did good work in a professional sense, having had a large and lucrative practice. He was deeply interested in hospital administration, and as one of the advisory committee of the *New York Herald* has been the means of directing public attention to and so effecting considerable improvements in the general administration of hospitals throughout the United States. Dr. Shrady's home life was delightful, and it was a great pleasure to see him surrounded by his grandchildren, who were devoted to him, and whom he used to keep amused for hours by the humour of his stories and the genuine affection which permeated the man for all young people. We have lost an old personal friend of many years standing, whom it was always a pleasure to meet, and we shall long mourn Dr. Shrady's loss, for he belonged to a type of men who are, unfortunately, rare in these days, for they are content to do genuine hard work for the good of the whole community unobtrusively and quietly. We tender our deepest sympathy to Mrs. Shrady and her children, whose loss must indeed be irreparable.

**The National Hospital for Paralysis.**

As already announced, several alterations and improvements are to be carried out at this hospital at a cost of £10,000. There is also much need for additional strength in the financial position of the hospital, which possesses a national character, and has a claim upon all thoughtful people who desire to identify themselves with the best kinds of hospital work. We congratulate the governors upon having secured the whole-hearted support of the Duchess of Albany, who, with the aid of the Jubilee Committee, has determined to make known to the nation the widespread benefits of the charity. A great effort is to be made to double the number of annual subscribers, which should not be a matter of difficulty if only the urgent importance to the whole community of an adequate provision for cases of paralysis and epilepsy can be driven home. Paralysis is not confined to any class, and attacks young and old alike, quite irrespective of their social position. In these hurrying, wearing times no one can say who may be the next victim to this dire disease, and we cannot understand any really intelligent person, who has the means to give and the knowledge to appreciate, who does not hasten to become identified with preventive work of this character by sending a liberal contribution to this hospital. Her Royal Highness the Duchess of Albany, as President of the Jubilee Committee, has made arrangements by which every contribution to the Jubilee Fund will be given to the hospital without any deduction whatever, the cost of printing, postage, etc., being borne by an Expense Fund instituted by the Duchess. H.R.H., in the course of a letter which is being widely circulated, declares she is deeply interested in the welfare of the

National Hospital, which is a memorial to her husband, and she is anxious that the proposed collections should be not only entirely successful from a financial point of view, but should, from their representative character, be a recognition of the national work of the hospital. Her Royal Highness hopes to raise the requisite sum by means of purses from all over the United Kingdom, and H.R.H. will be glad to hear at her address—Claremont, Esher—from any one who is willing to collect for a purse or otherwise assist. We wish this appeal a gratifying and complete success. The amount required is relatively small, and the whole sum should be readily raised without difficulty or delay. The Duchess of Albany has always been one of the most gracious and helpful members of the Royal family in every good work for the sick, and her daughter the Princess Alexander of Teck, as an active President of the League of Mercy, has done yeoman service in the same good cause. Everybody, therefore, must be attracted to an appeal which emanates from Her Royal Highness, and we hope many cheques of considerable amount for the National Hospital will speedily find their way to Claremont.

